# Geometrical Matching of SAR and Optical Images Utilizing ASIFT Features for SAR-based Navigation Aided Systems

**DOI:** 10.3390/s19245500

**Published:** 2019-12-12

**Authors:** Jakub Markiewicz, Karol Abratkiewicz, Artur Gromek, Wojciech Ostrowski, Piotr Samczyński, Damian Gromek

**Affiliations:** 1 Department of Photogrammetry, Remote Sensing and Spatial Information Systems, Faculty of Geodesy and Cartography, Warsaw University of Technology, 00-661 Warsaw, Poland; jakub.markiewicz@pw.edu.pl (J.M.); wojciech.ostrowski@pw.edu.pl (W.O.); 2 Institute of Electronic Systems, Faculty of Electronics and Information Technology, Warsaw University of Technology, 00-665 Warsaw, Poland; a.gromek@elka.pw.edu.pl (A.G.); P.Samczynski@elka.pw.edu.pl (P.S.); d.gromek@elka.pw.edu.pl (D.G.)

**Keywords:** SAR, Synthetic Aperture Radar, ASIFT, Despeckling Filter, Navigation, Structure from Motion, Iterative Closest Point

## Abstract

This article presents a new approach to the estimation of shift and rotation between two images from different kinds of imaging sensors. The first of the image is an orthophotomap that is created using optical sensors with georeference information. The second one is created utilizing a Synthetic Aperture Radar (SAR) sensor.The proposed solution can be mounted on a flying platform, and, during the flight, the obtained SAR images are compared with the reference optical images, and thus it is possible to calculate the shift and rotation between these two images and then the direct georeferencing error. Since both images have georeference information, it is possible to calculate the navigation correction in cases when the drift of the calculated trajectory is expected. The method can be used in platforms where there is no satellite navigation signal and the trajectory is calculated on the basis of an inertial navigation system, which is characterized by a significant error. The proposed method of estimating the navigation error utilizing Affine Scale-Invariant Feature Transform (ASIFT) and Structure from Motion (SfM) is described, and techniques for improving the quality of SAR imaging using despeckling filters are presented. The methodology was tested and verified using real-life SAR images. Differences between the results obtained for a few selected despeckling methods were compared and commented on. Deep investigation of the nature of the SAR imaging technique and noise creation character allows new algorithms to be developed, which can be implemented on flying platforms to support existing navigation systems in which trajectory error occurs.

## 1. Introduction

Over recent years, supporting navigation systems has become particularly important for several reasons. Firstly, the aim is to increase the precision of ammunition and flying objects. Secondly, it is necessary to create systems that can work without the support of GNSS (Global Navigation Satellite System). The lack of a GNSS (GPS, GLONAS, Galileo, or others) signal is a significant limitation whose probability of occurring increases due to potential international conflicts, as well as the possibility of the GNSS signal being jammed or interrupted. For these reasons, it is necessary to create independent systems that allow navigation in the absence of a satellite signal. One of the basic sensors that allows the navigation of objects is the inertial navigation system (INS). However, due to the significant drift and error increasing with time, inertial navigation requires additional systems. Currently, drift is reduced using GNSS systems; however, due to the limitations mentioned above, it is necessary to use additional sensors to support inertial navigation.

In the literature, several solutions have been proposed that allow the navigation of objects in the absence of a GNSS signal. The least effective solutions are purely vision methods, which are ineffective in the case of night missions, cloud cover, fog, smog, or smoke. Other remote sensing methods that do not use sensors working in the visible spectrum are used more often. Such solutions use sensors such as light detection and ranging (LIDAR) and an altimeter, which allows a terrain contour (DEM) to be obtained, and then compares the acquired contour with the data in the database. Such systems, also known as Terrain Contour Matching (TERCOM), are utilized to navigate unmanned aerial vehicles or cruise missiles in cases when a GNSS signal could be unavailable [1,2,3].

A recently developed technique is the use of Synthetic Aperture Radar (SAR) and Interferometric SAR (InSAR) radar for navigation correction [4,5,6,7]. Radio waves used in this technique can be easily used during cloud cover, night, and rain, which makes this method universal and independent of the weather conditions. In this case, a radar sensor and a database with georeferenced images are on board the flying platform. During the flight, the terrain is scanned by the radar and the obtained image is compared to the corresponding one in the database. Thanks to this, the inertial navigation error can be reduced.Such a concept of utilizing SAR and InSAR sensors to support on-board air platform navigational devices was proposed in the SARINA (SAR-based Augmented Integrity Navigation Architecture) project carried out during 2010–2012. The authors of this paper were also involved in this work, and as a result have developed a concept of the system and proved that SAR/InSAR sensors can be successfully used to support navigational devices. The results of this work were published by the authors in [4,5,6]. Initially, the concept was proved only using simulations at the technical readiness level (TRL) 3–4 in the nine-degree scale, where 9 denotes the system prototype after all the required certifications. In the previous work within the SARINA project, the merging of SAR/InSAR images with an optical image database was based on simple automatic shape recognition of the terrain targets, using target contours extraction. Image processing was then applied using different techniques, such as the Hough transform, to find specific targets and recognize their shape. These techniques were used for SAR image matches. For InSAR, algorithms based on matching 3D SAR interferograms with LIDAR elevation models were developed that were equipped with a simulated on-board database. The very promising results of the previous SARINA project motivated the authors to continue work on the topic, and to start developing a system on a higher TRL level. In the meantime, new algorithms on SAR and optical images have been developed and are widely used for other applications, such as in geodesy. The author intend to test efficiency of these techniques in cases so they could be used in support of air platform navigational devices. One such approach is based on the SIFT technique, and was presented by the authors in [7]. The novelty and usability of this approach is presented in [8]. The SIFT algorithm was used to find and match corresponding keypoints appearing in the SAR and optical images. This approach is extended and investigated in this paper by utilizing the more robust ASIFT (Affine Scale Invariant Feature Transform) method and its limitations. The novelty of the solution proposed and described in this paper is in the utilization of an innovative approach to the shift and rotation estimation between SAR and optical images. By finding characteristic points in both images and applying themodified version of the Structure from Motion (SfM) technique, an error can be estimated, which in turn provides the navigation drift correction in the flying platform.In the authors’ opinion, the concept of using the ASIFT technique is interest regarding the checking of the efficiency and precision of mismatched SAR and optical image calculations, which might be further used to correct platform navigation devices according to the algorithms developed by the authors in their previous work [4,5,6]. To the authors’ knowledge, an innovative approach to the shift and rotation estimation between SAR and optical images by also applying SfM techniques has not been used for navigation drift correction on a flying platform, on which the authors of this paper are currently working. In this paper, the authors present an overview of the SIFT and ASIFT techniques and their modifications, as well as the required pre-processing and its limits, which is taken into account when developing systems based on SAR systems for navigation drift corrections. The paper structures is as follows. In Section 2, there is a description of the proposed method presenting an overall problem characterization and solution. In Section 3, the basics of the SfM approach are presented to depict the main keys in this technique. Section 4 presents different types of SAR filters providing speckle noise reduction in images created using SAR radars. The results are presented in Section 5, and the conclusion closes the article. Additionally, the Appendix present the extensives results in a single part of the article. In the corresponding parts of the paper, there are references to the Appendix and images contained to ensure reader clarity.

## 2. Methodology

### 2.1. Overview of the Approach

The process of image registration refers to the alignment of two or more images of the same scene which might be obtained with the same sensor, time, and imaging conditions, as well as by different sensors and viewpoints. The process of orientating optical and SAR images is still an open issue, which creates many challenges [9,10,11,12,13,14,15,16,17,18,19]. Many issues can be dealt with in the process of the synergy of optical and SAR data, due to the great differences between passive and active remote sensing techniques. One of the issues is the problem with the speckle noise that influences the detection of robust corresponding/tie points [9,11,12,13,15,16,18,19]. Another issue is related to the differences in the image geometry acquired from these two devices [9,11,12,13,15,16,18,19]. Thus, many co-registration approaches have been proposed (e.g., [9,10,11,12,13,14,15,16,17,18,19]), but, in general, these approaches might be divided into two main categories: area- and feature-based methods. Nowadays, the Structure from Motion (SfM) methods, which are mostly based on the feature-based approach, are used for the co-registration of spaceborne SAR and optical images [9,11,12,13,15,16,18,19]. An extended description of the incremental SfM process is presented in Section 3. The classical SfM approach contains four main steps: (1) feature detection; (2) feature description; (3) descriptor matching; and (4) bundle adjustment. Due to the problems mentioned above, which influence the quality of detected and matched pairs of points, the SfM/co-registration approach has been modified by many authors. The first improvement is in the feature detection—downsampling SAR images or using the despeckling filters in the pre-processing step. Based on this way of pre-processing images, different types of features are detected such as blob, corners, or lines and segments. In the case of the feature description and matching, the modification is in the descriptor, or this step is eliminated and based only on the geometrical relationship. It should be stressed that these presented methods were validated in the spaceborne SAR and optical images.

The proposed methodology of the automation of SAR data registration with orthophotomaps as well as the SAR trajectory improvement, is a multi-stage process. This process is based on the original software and it consists of: (1) SAR data conversion to the raster form with a georeference file; (2) the aligning of orthophotomaps with a SAR raster based on the extended version of the ASIFT algorithm; (3) the relative orientation based on the classical SfM and modifiedIterative Closest Point (ICP) approach; (4) the analysis of the quality of the relative orientation of processed data; and (5) the final bundle adjustment.

In this investigation, the process of optical and SAR images was tested and validated with a high resolution SAR and orthophotomaps from altitude. It should be stressed that the entire investigation was performed on the full resolution of the images, and additionally tested on other pyramid levels. The authors decided to use a well-known SfM approach, but with the following modification: (1) the pre-processing of SAR data with different speckle noise reduction filters in order to increase the possibility of detecting and matching robustness corresponding points; (2) reducing the values of affine angle in the ASIFT algorithm—the values being related to the angle of the antenna; and (3) using a two-stage ICP procedure to eliminate the outliers and compute the correction parameters. Thanks to the use of the ASIFT algorithm, it was possible to detect well-distributed keypoints in the whole area under investigation. The authors decided to eliminate the description and matching step and replace it with the ICP alignment. This allowed keypoints to be treated as a point cloud, and minimize the distance between these two point clouds in an iterative process with the simultaneous correction of translation and rotation of SAR data.

**Figure 1:**
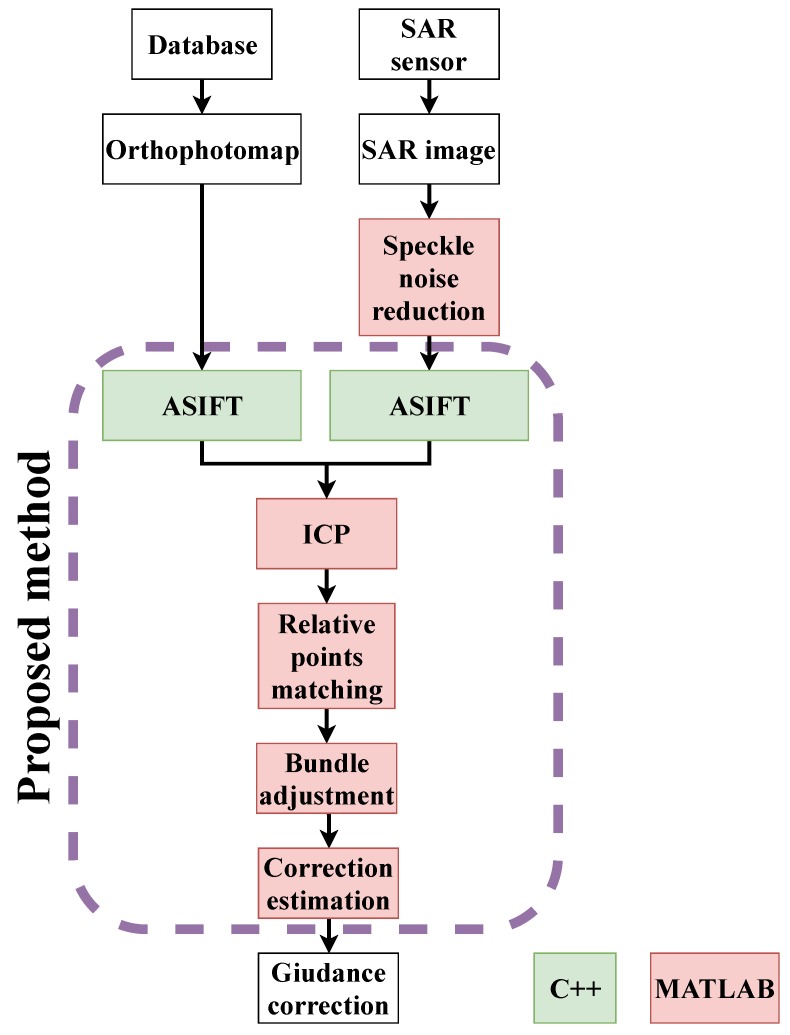
Diagram of the performed research: Processing and orientation of SAR images and orthophotomaps.

In Figure 1, a diagram of the performed research work and experiments is shown. To perform a complete analysis of the possibility of applying the modified version of the registration algorithms for the detection and matching of correspondence points, a combination of these should be determined to obtain the best results. Verification of the following parameters are required:

1. The keypoints distributions on SAR images and orthophotomaps.
2. The influence of the SAR image correction on the quality and number of the keypoints.
3. The number of detected keypoints used in the final cooregistration process.
4. The orientation accuracy of marked check-points.

The idea of the automatic SAR data registration and trajectory improvement presented in this paper (according to the diagram presented in Figure 1) consists of the following steps:

1. Generation of the SAR raster with georeferences based on the trajectory information.
  This step is one of the most important parts in the whole automatic registration process. It influences both the computation time and convergence of the ICP process.
2. *Pre-processing of SAR data to reduce the influence of the noise.*
  - RAW data
  - Multilook-2D filter
  - Averaging filter
  - Minimum Mean Square Error filter
  - Enhanced Lee filter
  - Gamma MAP filter
  - SAR Block Matching 3D filter
3. Selection of the part of the orthophotomap which approximately covers the SAR image, based on the SAR footprint extended by 50 m in each direction.
4. *Detection and matching of the keypoints using the modified ASIFT algorithms in the SAR images and orthophotomaps:*
  4.1. Detection of keypoints in RAW SAR images on three pyramid levels (full resolutions, as well as **1/2**, 1/4, and 1/16 of the full resolution of the raster)—ASIFT algorithm.
  4.2. Detection of keypoints in SAR images with the speckle noise reduction parameters: 1, 4, 8, and 16 levels (full resolution, and **1/2**, 1/4, and 1/16 of the full resolution of the raster)—ASIFT algorithm.
  4.3. Detection of keypoints on orthophotomap levels (full resolution, as well as **1/2**, 1/4, and 1/16 of the full resolution of the raster)—ASIFT algorithm.
  4.4. Matching keypoints by the incremental ICP method:
    - Approximate registration with 20 iterations and linear threshold deviation 20 m.
    - Removal of orthophotomap keypoints that are outside the SAR area.
    - Registration with 10 iterations and linear threshold deviation 5 m.
    - Removal of the SAR point outliers based on the ${\mathrm{RMSE}}_{xy}$.
    - Final bundle adjustment.
5. Analysis of the quality of data registration on the marked check-points.

The presented SAR data registration processing is an original approach, and for this reason original applications were applied (based on the OpenCV library and MATLAB software).

The presented technique was intensively tested, validated, and compared with the methods existing in the literature. Especially two approaches were investigated [9,17]. However, the method described in [17] is inefficient and fails due to the speckle noise on SAR images. The approach presented in [9], in turn, is accurate only on urban areas, and such assumption cannot be applied in the considered case. The authors main goal was to provide universal approach able to work in both, urban and rural areas.

## 3. The Principles of the Structure from Motion

Modern software packages, application, and function libraries dedicated to raster data orientation, and 3D shape reconstruction utilize algorithms based on a combination of methods commonly applied in Computer Vision (CV) and conventional photogrammetric approaches. These types of algorithms and methods allow the geometry and appearance of an object or an entire scene to be captured, and have been used in video games assets [20], virtual tours [21], virtual and augmented reality [22], and cultural heritage [23,24,25,26,27], among others. One of the most important approaches is the Structure from Motion (SfM) method [20,27,28,29]. The SfM pipeline allows for the reconstruction of three-dimensional structures based on a series of images (rasters) acquired from different positions (observation points) [20].

**Figure 2:**
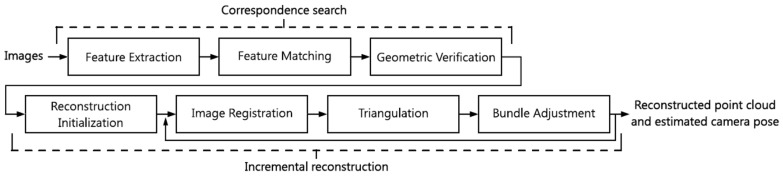
Incremental SfM pipeline [20].

Figure 2 shows the overview of the incremental SfM workflow, which contains the following steps: (1) feature extraction; (2) feature matching; (3) geometric verification; (4) reconstruction initialization; (5) image registration; (6) triangulation; and (7) bundle adjustment. To generalize, the SfM approach might be divided into two main parts: the correspondence search phase (1–3) and iterative reconstruction phase (4–6). Based on these two phases, the estimation of the camera position for each image as well as a 3D reconstructed tie point, called a spare point cloud [20], can be done. In this article, only the correspondence search with the computation of registration parameters is described.

### 3.1. The Feature Extraction

The feature extraction process is the first step of the SfM pipeline, which is based on detectors. For each image (raster data) given as an input, a group of characteristic points (called keypoints) are detected (excreted) based on the local characteristic of the image intensity. For feature extraction, different methods and algorithms can be used which affect the robustness of the detected features, as well as the efficiency of the matching method. Nowadays, two types of commonly used algorithms are corner (such as FAST, Harris, etc.) and blob detectors (i.e., SIFT and its modification) [30,31,32,33,34,35,36,37,38]. Brief summaries of studies into data fusion and finding correspondences between SAR and optical images have been recently made by [39] and [14]. Due to the fact that many feature detectors exist, in this section, only SIFT [40] and ASIFT (a modified version of the SIFT) [38] algorithms are focused on, which were used in this investigation.

The SIFT (Scale Invariant Feature Transform) algorithm, which was originally proposed by Lowe [40] for the registration of optical images, has already been adapted for the matching of SAR images. In their studies, [41] focused on the denoising of SAR data with curvelet transformation and the evaluation of SIFT performance on SAR images for various terrain types. The speckle noise, which is characteristic for SAR images, has a vast influence on the SIFT algorithm’s performance, and beside denoising (which is further described in Section 4) deeper modification into SIFT feature extraction has also been proposed in the SAR-SIFT approach [19]. However, the noise is not the only difference between SAR and optical images; geometrical differences also have an influence on image registration [14]. In this research, the authors are searching for corresponding points between aerial SAR images where image points are recorded according to the object-to-antenna distance and optical orthophotomaps where bare ground is in ortho projection, but all other objects that are elevated from the ground (e.g., buildings or vegetation) are distorted (shifted) by central projection from the optical image. To overcome these geometric distortions, the authors propose to use the ASIFT (Affine Scale Invariant Feature Transform) [38], which is a modification of the SIFT algorithm. The main idea of the ASIFT algorithm is to simulate a set of sample views of the initial images, obtainable by varying the two camera axis orientation parameters, namely the latitude and the longitude angles, which are not detected by the classical SIFT method [38]. Then, the SIFT method is applied to all the virtual images generated. Thus, ASIFT covers all six parameters of the affine transform and guarantees full affine invariant independence. In the ASIFT algorithm, each image is transformed by simulating all possible affine distortions caused by the change of the initial camera positions. To perform this, the camera model as well as the affine model are utilized (Equations (2) and (3)):

(1) $$u=S_{1}G_{1}ATu_{0},$$

where *u* is a digital image; $u_{0}$ is an (ideal) infinite resolution frontal view of the flat object; *T* and *A* are, respectively, a plane translation and a planar projective map due to the camera motion; $G_{1}$ is a Gaussian convolution modeling the optical blur; and $S_{1}$ is a standard sampling operator on a regular grid with mesh 1.

(2) $$u\left(x,y\right)\to u\left(ax+by+e,cx_{d}y+f\right)$$

(3) $$A=\left[\begin{array}{cc} a & b\\ c & d \end{array}\right]=H_{\lambda }R_{1}\left(\psi \right)T_{t}R_{2}\left(\phi \right)=\lambda \left[\begin{array}{cc} \cos\psi & -\sin\psi \\ \sin\psi & \cos\psi \end{array}\right]\left[\begin{array}{cc} t & 0\\ 0 & 1 \end{array}\right]\left[\begin{array}{cc} \cos\phi & -\sin\phi \\ \sin\phi & \cos\phi \end{array}\right]$$

where λ>0 is the determinant of *A*, $R_{i}$ are rotations, ϕ∈[0,π), and $T_{t}$ is a tilt, namely a diagonal matrix with first eigenvalue t>1 and the second one equal to 1.

It is possible to prepare the decomposition of the camera motion parameters into the viewing point angels (longitude (ϕ) and latitude ($\theta =\arccos\frac{1}{t}$)), spin of the camera (ψ) and zoom factor (λ). In the ASIFT algorithm, images undergo rotation with the angle ϕ, which is represented by the tilt parameter $t=\frac{1}{\cos\theta }$. In the ASIFT algorithm, the influence of the tilt (latitude rotation) is performed by the t-sampling and the Gaussian convolution with standard deviations $c\sqrt{t^{2}-1}$ (c=0.8). It is assumed that the latitudes θ are sampled as the geometric series $1,a,a^{2},\ldots ,a^{n}$ with a>1 and choosing $a=\sqrt{2}$ is a good compromise between the accuracy and the number of steps. The authors of the ASIFT algorithm proposed the *n* value equals 5, which results in the tilt being simulated 32 times [38]. In the case of the longitudes ϕ, the arithmetic series $0,\frac{b}{t},\ldots ,\frac{kb}{t}$ with the $b≅\frac{2\pi }{5}$ and $k\frac{b}{t}<\pi$ is used. After the process of virtual image generation (which includes the skew, tilt, and rotation), any detector, such as SIFT or SURF, might be used. In this study, the SIFT detector was used.

### 3.2. The Feature Description

After the process of detecting characteristic points, the next step is to describe this by analyzing the nearest points. In the literature, several descriptors are presented such as SIFT, SURF, Daisy, etc. [42], but the authors decided to describe only the SIFT detector, which was used in this study. The main idea of the SIFT descriptor is to compute the local image gradients at the selected scale in the region around the tested keypoint. The full description of the descriptor can be found in Lowe’s publication [34], and a further SAR-specific modification of this descriptor can be found in [19]. In the original ASIFT algorithm proposal, the SIFT detector is used. This mathematical computation allows one to determine which detected key-point and its surrounding is highly distinctive yet as invariant as possible to remaining variations, such as changes in illumination or 3D viewpoint. The descriptor calculation is similar to determining the detector as the image gradient magnitude and orientations are sampled around the keypoint localization for each octave and each Gaussian blur.

### 3.3. The Feature Matching and Images Registration

The detection and description of features for each characteristic point are important components in the process of the detection of conjugate points in digital images. To determine if the keypoint (obtained through the point detection and description process) might be threaded as a tie point, the feature matching process is used. This allows one to take into consideration two points from different images, but which are characterized by the same description as a homologous point. Different strategies can be used for effectively computing matches between images, but two which are usually used are: approximate nearest-neighbor-based point matching [43] and brute-force matching [43]. In the nearest neighbor approach, the points are stored in the k-dimensional space (k-d tree structure). This allows one to compute the nearest neighbors based approximately on the minimal distances between the descriptor values [43]. The brute-force matcher is much simpler because it takes the descriptor of one feature in the first set and matches it with all other features in the second set, using a set of distance calculations. As a result, the closest feature is returned. The feature matching based only on the descriptors is justified in the case of the sensors with a similar wavelength and images obtained from the same optical system. In the case of matching heterogeneous images (e.g., optical and SAR), where a large amount of outliers is expected during feature (descriptors) matching, also adding additional geometrical constraints could be beneficial. [18] used spatial consistent matching, which assumes that the geometrical relationship between matching features should not change too much across images. However, this solution is still largely based on SIFT-like descriptors which are constructed using gradients of image values that can differ significantly between SAR and optical images because of radiometric differences [39]. To overcome this problem, the authors of this paper proposed another way of keypoint matching, where the feature matching is based solely on the geometrical relations between keypoints and utilizes the Iterative Closest Point (ICP) algorithm [44]. The ICP algorithm is a well-known algorithm, implemented in many commercial and open-source software, as well as in programming libraries (such as VTK, open3D, and PCL) [45,46,47] and is used for oriented point clouds—the minimal distance between two point clouds [44]. There are many variants of the ICP algorithm [44,48,49,50,51,52]; however, in this section, only one of them (thebasic one), proposed by Besl and McKey, is described in more detail.

When considering two datasets, it is possible to determine interrelations between them expressed by the phenomenon:

(4) $$y_{i}=Rx_{i}+y_{0}$$

where *R* is a rotation matrix, $x_{i}$ are the point coordinates in the input point cloud reference system, and $y_{0}$ is the translation vector. In the proposed approach, only 2D coordinates are used because orthophotos do not contain height information, and SAR images during pre-processing are projected onto a plane (with mean height). Real-world coordinates can be easily obtained for orthophotomaps because they are georeferenced, and, for SAR images, coordinates are estimated with direct georeferencing using the plane trajectory from GNSS and/or an inertial navigation. The main objective of the ICP method is to align two point clouds, based on shapes or models, by using the Euclidean distance dependence between the nearest point from the initial set of points and the reference. For this purpose, based on the least square method using the distance square minimization (Equation (5)) function, transformation parameters are calculated for points on the areas for which common coverage occurs.

(5) $$e^{2}=\sum_{i}^{}{∥Rx_{i}+y_{0}-y_{i}∥}^{2}\Rightarrow \min.$$

In each of the iterations of the ICP algorithm, the transformation can be determined using the four main methods: SVD decomposition [53], Hora quaternions [54], Horn’s orthogonal matrix [55], and based on Walker’s double quater [56]. These algorithms are characterized by similar effectiveness and stability of operation in the case of noisy point clouds [57]. Based on the calculated translation and rotation parameters, the initial point cloud is transformed and the whole process repeats until the minimal distance threshold is not reached. The presented ICP method is commonly used for a 3D point cloud registration. However, in the case of the keypoint matching, only 2D space computation is performed. When using the ICP method, it is important to have a good first approximation of the relative orientation parameters, because without it the solution of the final registration might fail. In the proposed method of the SAR images and orthophotomaps coregistration, this condition is met thanks to the approximate georeference of both data sources. This way of keypoint matching allows one to reduce the problem of the influence of the descriptor, because it is only based on the geometrical relationship between the keypoints detected in SAR and optical images, and could be easily used as long as the approximate georeference of both images are known. One of the most important parts of the image orientation is the geometric verification of the matched keypoints. This correct keypoint matching determines the final correctness of the alignment and quality of the data registration. Depending on the methodology of keypoint matching—descriptor matching or keypoint ICP matching—different methods are used, but overall it should be stressed that the feature matching phase only verifies pairs of points on matched images. Considering the descriptor matching, it is not guaranteed that the matches found actually correspond to 3D points in the scene, and outliers could be included. It is important to find the correct geometric transformation that correctly maps the corresponding point. Using the descriptor method, it is necessary to choose the correct mathematical model for transformation, i.e. homography or similarity transformation. A list of commonly used methods are presented in [20]. To reduce the outliers from the data, it is necessary to implement robust estimation techniques such as RANSAC (Random Sample Consensus [56]) or MLESAC (Maximum Likelihood Estimation SAmple and Consensus), which is a generalization of the RANSAC algorithm [58,59]. In the case of the ICP matching method, the transformation method is predefined. However, for eliminating the outliers, a transformation method such as a similarity transformation can be applied. In this investigation, the registration parameters from the keypoint ICP method are treated as final and applied to the correction of the SAR georeference.

## 4. SAR Image Preprocessing

Because all kinds of algorithms which detect keypoints such as ASIFT/SIFT/SURF work on intensity images, the presence of speckle noise in SAR images is an obstacle. Speckle noise affects the behavior of keypoint detector algorithms, which is why it has to be reduced. *Speckle noise* is a common phenomenon that accompanies all coherent imaging systems, such as SAR sensors [60]. The source of this “noise” is attributed to random interference between the coherent returns issued from the numerous scatterers present on the surface of a scene, in relation to the wavelength of incident radar wave. The resulting *speckle* has a multiplicative nature, thus SAR imagery is characterized by strong intrinsic noise (hereafter, referred to as *speckle* only). Typically, for a single-look SAR image, **ISNR** (Intrinsic Signal to Noise Ratio) = 0 dB, and we have the same amount of signal and noise power/level. It appears in the image as strong fluctuations in its brightness, hardening the image interpretation.

**Figure 3:**
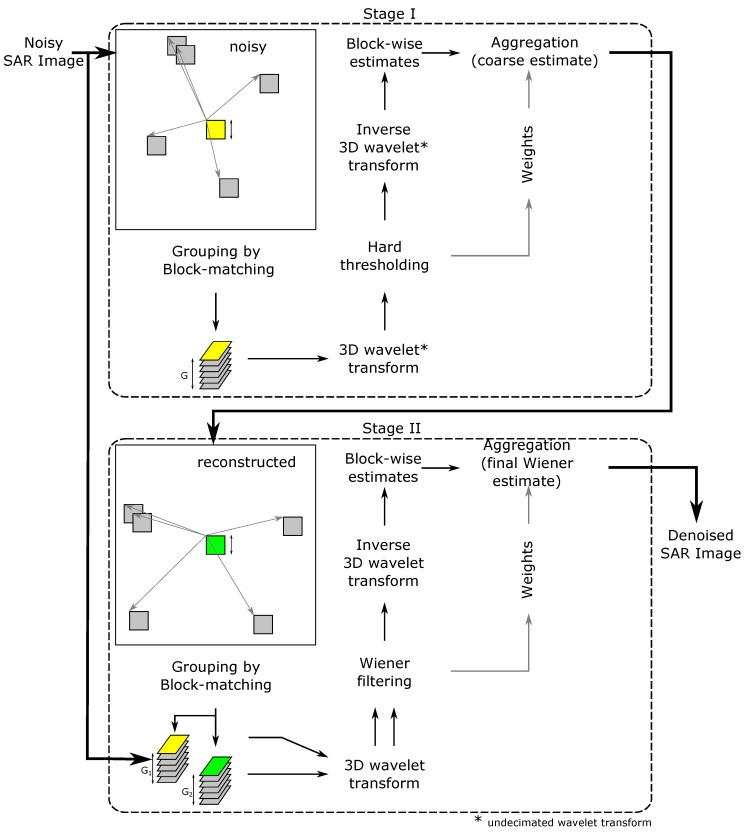
SAR-BM3D despeckling filter block diagram.

As described in the literature, utilizing SAR radar images for different purposes often requires additional operations, increasing SNR. If the operations are not performed, the results can be strongly disturbed [7,61]. Some despeckling methods should be taken into account in a processing pipeline, even though they might be computationally complex (for example as shown in Figure 3), because by reducing the influence of noise, better quality results can be obtained, which might be critical in many applications. In the presented approach, speckle noise plays an important or even key role in the final outcome. Despite the resolution reduction caused by the filter usage, the local dynamic of the image is significantly improved, which allows further processing to be carried out. Further processing in this case means keypoint localization, which is the finding of characteristic points in the image. If the speckle noise is present, disturbed pixels may be considered as keypoints even if they are only distorted.

**Figure 4:**
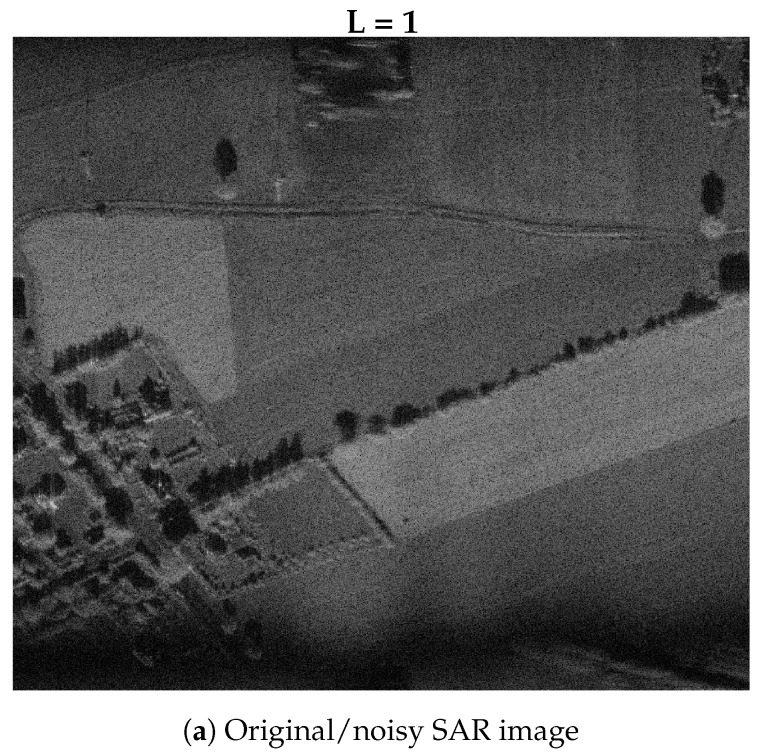

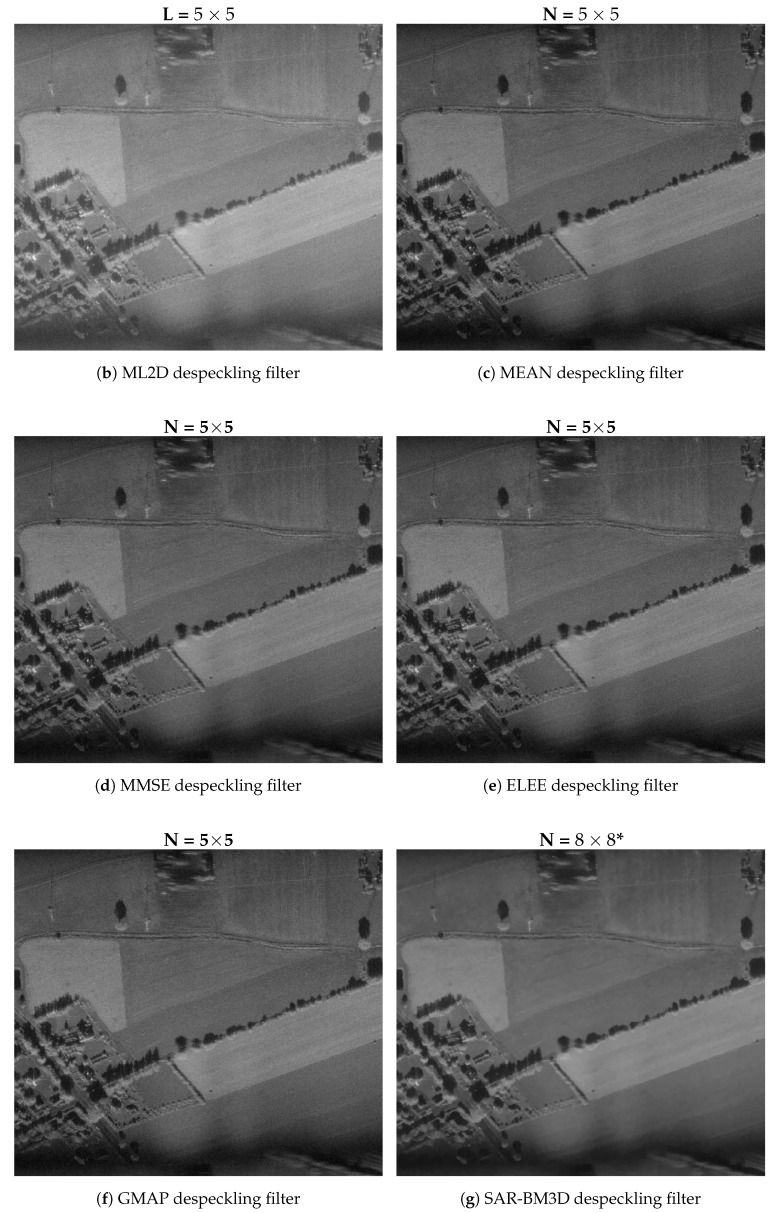
SAR imagery filtration results: *L* is the number of (multi)looks and *N* is the filter window size (equiv. to *L*). * Even window size forced by UWT transform implementation.

For a fully developed *speckle* (see Figure 4a), the brightness fluctuations in SAR images can be modeled using a gamma distribution (Equation (6)). The gamma distribution is one of the basic types of distributions used in SAR radar imaging (although not the only one [60]):

(6) $$\begin{array}{cc} p_{z}\left(z,n|\sigma ,L\right) & =\frac{n}{\Gamma (L)}{\left(\frac{L}{\sigma }\right)}^{L}z^{nL-1}\;\exp(-\frac{Lz^{n}}{\sigma }),\\ n & =1\;\mathrm{for}\;\mathrm{intensity}\;\mathrm{data},\;I=A^{2}\\ n & =2\;\mathrm{for}\;\mathrm{amplitude}\;\mathrm{data},\;A, \end{array}$$

with the following statistical moments:

(7) $$E\left[z^{m}\right]=\frac{\Gamma (L+m/n)}{\Gamma (L)}{\left(\frac{\sigma }{L}\right)}^{m/n},$$

where *z* is the given pixel value; *n* is the amplitude (*A*) or intensity ($A^{2}$) data format; σ is the expected (true) pixel value; *L* is the number of (multi)looks/averages; and *m* is the statistical moment order (m=1 is the mean value; m=2 is the mean square value; etc).

From Equations (6) and (7), providing n=1, the basic maximum likelihood estimators (MLE) can be determined:

(8) E[z]=〈z〉,

(9) $$ENL\;\left(\equiv L\right)=\frac{E^{2}\left[z\right]}{E[z^{2}]}=\frac{{\langle z\rangle }^{2}}{{\langle z-\langle z\rangle \rangle }^{2}},$$

where E[•] is the expected value (operator); 〈•〉 is the arithmetic averaging; and ENL or ENIL is the equivalent number of (independent) looks.

One of the basic ways to deal with the problem of high *speckle* noise level is the pre-processing of SAR imagery with despeckling filters. Commonly, a classical multilooking technique is applied. Nevertheless, there are plenty of filtration/despeckling algorithms.The authors decided to describe types of filters which were used in experimental processing since the results are different for each filter type.

### 4.1. ML2D: Multilook–2D Filter

In contrast to the classical multilooking procedure [62], Multilook–2D (which might also be called a non-coherent version of the multilook procedure) works in both image dimensions, i.e. range and cross-range (X and Y) [61]. Thanks to this, a more effective *speckle* reduction can be made with less degradation of the image spatial resolution at the same time.

The idea for the 2D multilooking procedure was taken from optical image processing. The algorithm operates on the entire, already prefocused SAR image, based on Fourier domain processing.

(10) $$\begin{array}{cc} Z\left\{\omega_{x},\;\omega_{y}\right\} & =F_{2D}\left[z(x,\;y)\right],\\ Z\left\{\omega_{x},\;\omega_{y}\right\} & \approx \sum_{i=1}^{L}Z_{i}\left\{\omega_{x}^{\prime },\;\omega_{y}^{\prime }\right\},\\ \tilde{z}\left(x,\;y\right) & =\sum_{i=1}^{L}\;\left|\;F_{2D}^{-1}\left[\;Z_{i}\left\{\omega_{x}^{\prime },\;\omega_{y}^{\prime }\right\}\cdot W\left\{\omega_{x}^{\prime },\;\omega_{y}^{\prime }\right\}\;\right]\;\right|, \end{array}$$

where z(x,y) is the original noisy SAR image; $\tilde{z}\left(x,\;y\right)$ is the *despeckled* (reconstructed) SAR image; $Z\left\{\omega_{x},\;\omega_{y}\right\}$ is the two-dimensional Fourier spatial frequency spectrum $\left\{\omega_{x},\;\omega_{y}\right\}$; $Z_{i}\left\{\omega_{x}^{\prime },\;\omega_{y}^{\prime }\right\}$ is the partial two-dimensional Fourier spatial frequency spectrum $\left\{\omega_{x}^{\prime },\;\omega_{y}^{\prime }\right\}$; $W\left\{\omega_{x}^{\prime },\;\omega_{y}^{\prime }\right\}$ is the window (spectrum weighting) function; and *L* is the number of (multi)looks...

The algorithm transforms the entire radar image into a two-dimensional Fourier space. Then, it divides the spectrum into *L* sub-bands (with the possibility of overlapping, typically ≤50%) and filters each sub-band by appropriate weighting. Finally, it reconstructs the image throughout, returning to the spatial domain for each sub-band, generating sub-pictures, and incoherently putting all of them together. The resulting (reconstructed) SAR image is characterized by its reduced *L*-times speckle level. Undesirable side effects are $\sqrt{L}$-times spatial resolution degradation and visible side lobes resulting from spectrum windowing. The result of filtering a real-life SAR radar image is shown in Figure 4b.

### 4.2. MEAN: Averaging Filter

One of the simplest noise filtration techniques is averaging image samples over the area around a pixel, combined with a sliding window technique (see Equation (11)). For a gamma distribution [60], the averaging operation also corresponds to the maximum likelihood estimator (MLE) [63].

(11) $$\tilde{z}\left(k\right)=\frac{1}{N}\sum_{i=1}^{N}z\left(k+i\right),$$

where z(k) is the original noisy SAR image; *k* is the linear image pel index (k=x+Width∗y) *Width* is the image width in pixels/pels; *Height* is the image height in pixels/pels; and x,y is the image pixel(s) indexes (horizontal and vertical); $\tilde{z}\left(k\right)$ is the *despeckled* (reconstructed) SAR image; *N* is the filter window size in pixels/pels, e.g. 3×3, 5×5, etc; and *i* is the filter window linear index ∈〈1,N〉.

As a result of the averaging filtration, the speckle standard deviation is reduced by a factor of $\sqrt{N}$-times, where *N* is the number of pixels in the filter window. Note that in extreme cases, if the window size is N=1 (only the central pixel without its surroundings will be taken into account), no filtration effect will be noticed. In turn, for a large window size N, the speckle will be reduced at a cost of image detail degradation. An averaging filter that does not include local image statistics results in severe degradation of details (such as: lines, edges and point target blurring). To overcome this issue, only suitably sized windows should be chosen (small windows are usually used e.g. 3×3, 5×5 points in two dimensions). The result of filtering a real-life SAR radar image with an averaging filter is shown in Figure 4c.

### 4.3. MMSE: Minimum Mean Square Error Filter

The above-mentioned despeckling filters fail when the assumption of constant pixel value within the filter window breaks down. The filter should then adapt to take account of excess fluctuations compared to *speckle* within the analysis window. One approach to such an adaptive filter is to provide a model-free minimum mean-square error (MMSE) filter based on measured local statistics. The solution of minimizing the mean square error for a pixel $\tilde{z}\left(k\right)$ is to perform first-order expansion about its local mean value $\bar{z}\left(k\right)$ (Equation (11)) so that:

(12) $$\begin{array}{cc} \tilde{z}\left(k\right) & =\bar{z}\left(k\right)+\alpha \cdot \left(z\left(k\right)-\bar{z}\left(k\right)\right),\\ \alpha & =\frac{{\bar{V}}_{\sigma }}{{\bar{V}}_{z}}=\frac{{\bar{V}}_{z}-1/L}{{\bar{V}}_{z}\;\left(1+1/L\right)}, \end{array}$$

where z(k) is the original noisy SAR image; *k* is the linear image pel index (k=x+Width∗y); $\bar{z}\left(k\right)$ is the expected/mean pixel value (see Equation (11)); $\tilde{z}\left(k\right)$ is the *despeckled* (reconstructed) SAR image; α is the linear interpolation weight; and ${\bar{V}}_{z}$ is the normalized variance.

As can be seen, it is a weighted sum (or linear interpolation) between the mean and given/current pixel value. In cases when there is no fluctuation coming from the image texture, the weighting factor α→0 and the pixel value is assigned the average value of its surroundings. On the other hand, when the fluctuation of the image texture takes on a significance weighting factor $\alpha \to \frac{L}{L+1}$, the value of the pixel will be scaled by a factor of alpha—which can happen in places where there are lines, edges, or any other texture/spatial features. The result of filtering a real-life SAR radar image with an MMSE filter is shown in Figure 4d. A similar approach was presented by Lee [64,65].

### 4.4. ELEE: Enhanced Lee Filter

An approach presented by Lee [64] considers an optimal linear filter that is equivalent to a first-order Taylor expansion of the multiplicative noise model $z\left(k\right)=\bar{z}\left(k\right)\cdot \eta$ about expected $\bar{z}\left(k\right)$ and *speckle* component η. Multiplicative noise can be rewritten as an additive one by $z\left(k\right)=\bar{z}\left(k\right)+\left(\eta -1\right)\cdot \bar{z}\left(k\right)$, thus resulting in a similar form to MMSE (Equation (12)), but the weighting factor α is now given a bit differently:

(13) $$\begin{array}{cc} \tilde{z}\left(k\right) & =\bar{z}\left(k\right)+\alpha \cdot \left(z\left(k\right)-\bar{z}\left(k\right)\right),\\ \alpha & =\frac{{\bar{V}}_{z}-1/L}{{\bar{V}}_{z}}, \end{array}$$

where z(k) is the original noisy SAR image; *k* is the linear image pel index (k=x+Width∗y); $\bar{z}\left(k\right)$ is the expected/mean pixel value (see Equation (11)); $\tilde{z}\left(k\right)$ is the *despeckled* (reconstructed) SAR image; α is the linear interpolation weight; and ${\bar{V}}_{z}$ is the normalized variance.

When there is no image texture variation, it would be expected that the estimate ${\bar{V}}_{z}$ is close to pure *speckle*, i.e. 1/L, so that α→0 and $\tilde{z}\left(k\right)=\bar{z}\left(k\right)$ (same as for the MMSE algorithm). However, texture variability causes ${\bar{V}}_{z}$ to be different from the *speckle*. If pixel value z(k) is sufficiently large compared with its surroundings, it yields a large value of ${\bar{V}}_{z}$, so that α→1 and $\tilde{z}\left(k\right)=z\left(k\right)$, and the pixel value remains unchanged (no filtration effect). Thus, the response of Lee’s filter to strong targets differs from MMSE in that it ignores the *speckle* contribution to the target when making the filtration, corresponding to treating the bright pixel as a point target that would not give rise to *speckle* fluctuations. The result of filtering a real-life SAR radar image with an enhanced Lee filter is shown in Figure 4e.

### 4.5. GMAP: Gamma MAP Filter

The Gamma filter is a Maximum A Posteriori (MAP) filter based on a Bayesian analysis of the image statistics. It assumes that both the SAR image texture and the *speckle* follows a Gamma distribution (Equation (6)). The imposition of these distributions yields a K-distribution [66], which is recognized to match a large variety of different types of radar clutter, such as land and ocean type cover. The formula of the GMAP filtration is given by:

(14) $$\begin{array}{cc} \tilde{z}\left(k\right) & =\frac{\bar{z}\left(k\right)\cdot \left(\nu -L-1\right)+\sqrt{{\bar{z}}^{2}\left(k\right)\cdot {\left(\nu -L-1\right)}^{2}+4\cdot \nu \cdot L\cdot z\left(k\right)\cdot \bar{z}\left(k\right)}}{2\cdot \nu },\\ \nu & =\frac{1}{{\bar{V}}_{\sigma }}=\frac{1+1/L}{{\bar{V}}_{z}-1/L}, \end{array}$$

where z(k) is the original noisy SAR image; *k* is the linear image pel index (k=x+Width∗y); $\bar{z}\left(k\right)$ is the expected/mean pixel value (see Equation (11)); $\tilde{z}\left(k\right)$ is the *despeckled* (reconstructed) SAR image; ν is the texture model order parameter; and ${\bar{V}}_{z}$ is the normalized variance.

In the case of pure *speckle*, it would be expected that ${\bar{V}}_{z}=1/L$ so that ν→∞ and $\tilde{z}\left(k\right)=\bar{z}\left(k\right)$ (as with the MMSE and Lee filters). However, as mentioned above, texture variability causes the estimate ${\bar{V}}_{z}$ to be different from the *speckle*. In this case, excess pixel value z(k) is understood as image texture contribution, and the parameter takes small values, thus the Gamma MAP estimator weights the current pixel value by the properly calculated weight $\tilde{z}\left(k\right)=z\left(k\right)/\left(1+1/L\right)$. The result of filtering a real-life SAR radar image with Gamma MAP filter is shown in Figure 4f.

### 4.6. SAR-BM3D: SAR Block Matching 3D Filter

Among different types of despeckling filters [64,67,68,69], one in particular demonstrates great improvements in speckle filtration performance—SAR-BM3D [70]. The filter has a very complicated structure; however, to outline the processing flow, it can be summarized as shown in Figure 3. The filter is divided into two stages, the first of which makes a coarse estimate of the image content/texture by Non-Local Meanings (NLMs), and the second stage makes a fine estimate of the image texture by Wiener filtration. The algorithm operates in the wavelet transform domain, and the general formula of SAR-BM3D filtration is given by:

(15) $$\begin{array}{cc} \tilde{Z}\left(k\right) & =\bar{Z}\left(k\right)+\frac{V_{\Sigma }^{2}\left(k\right)}{V_{\Sigma }^{2}\left(k\right)+V_{U}^{2}\left(k\right)}\cdot \left[Z\left(k\right)-\bar{Z}\left(k\right)\right],\\ Z(k) & =\Sigma \left(k\right)\cdot H=\Sigma \left(k\right)+\left(H-1\right)\cdot \Sigma \left(k\right)\\ & \;\;\;=\Sigma (k)+U(k),\\ Z(k) & ={\mathrm{WT}}_{3D}\left[z(k)\right],\;\tilde{z}\left(k\right)={\mathrm{WT}}_{3D}^{-1}\left[\tilde{Z}\left(k\right)\right], \end{array}$$

where Z(k) is the original noisy SAR image in wavelet transform domain, and capitalized letter means a transformed value (e.g., X=WT[x] ); $\bar{Z}\left(k\right)$ is the expected (true/original) image texture in WT transform domain; $\tilde{Z}\left(k\right)$ is the *despeckled* (reconstructed) SAR image in WT transform domain; u(k) is the zero mean, additive signal dependent *speckle* noise ($u\left(k\right)=\left(\eta -1\right)\cdot \bar{z}\left(k\right)$); *k* is the linear image pel index (k=x+Width∗y); and η is the fully developed *speckle* noise.

The first stage comprises three steps:

- grouping—for each reference block, the most similar blocks are grouped together;
- collaborative filtering—each 3D group undergoes UWT(Undecimated Wavelet Transformation); hard thresholding, and inverse UWT; and
- aggregation—all filtered blocks are suitably weighted, giving a coarse estimate of the image.

The second stage also comprises the same three stages, but with the following differences:

- grouping—blocks are located based on the coarse estimate provided by the first stage;
- collaborative filtering—each 3D group (of noisy blocks) undergoes WT((Decimated) Wavelet Transformation); Wiener filtering, and inverse WT; and
- aggregation—as in Stage 1, giving a final estimate of the image.

The presented filtration algorithm comprises several state-of-the-art techniques, thus giving better performance in terms of signal-to-noise ratio and perceived image quality than any of the other aforementioned filters. The result of filtering a real-life SAR radar image with the SAR-BM3D filter is shown in Figure 4g.

### 4.7. Filtration Performance

In this subsection, SAR image despeckling filtration results are presented and compared. The image used is shown in Figure 4a; the imaged area is located near Plock city/refinery, Poland. The image was made by WUT’s (Warsaw University of Technology) proprietary radar imaging system. The images shown in Figure 4 present results for different filters applied. In Table 1, a summary of the despeckling filtration performance is presented.

It should be emphasized that the implementations of filters were made as so-called MATLAB ® *m*-scripts, and the entire processing chain was made in this computing environment. Thus, the filtration performance in terms of absolute processing time is not meaningful. However, it reflects the computational complexity for each filter.

From the results presented in Table 1 and Figure 4, it can be seen that SAR-BM3D is the most effective filter. In this case, the equivalent number of looks (ENL) indicator is many times higher than for the others; also the visual assessment is outstanding (see Figure 4g). However, at the same time, it is the slowest one; its execution time is several times longer compared to the other filters, which is a consequence of its sophisticated/complex signal processing pipeline. However, there is a potential to significantly speed up its execution time (e.g., by using Cuda).

On the other hand, the ML2D despeckling filter provides good performance for both *speckle* reduction and execution time (see Table 1 and Figure 4b). In comparison with the other filters, it performs the fastest, giving very good image quality (ENL≈L is the desired number of multilooks). Nevertheless, this statement does not disqualify the usability of the other filters.

The ENL indicator is not fully unambiguous in the context of radar to optical image comparison. It is important to preserve details of the unique structural features of the image resulting from the texture of the observed scene. Thus, an assessment based solely on ENL is not conclusive. Therefore, in the following sections, further analysis is carried out for all the filters described above.

## 5. Results

**Figure 5:**
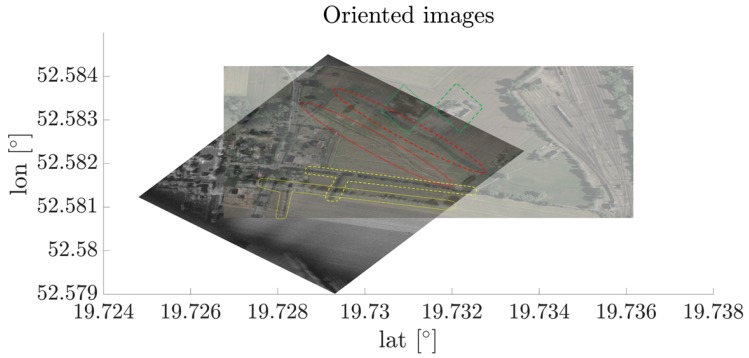
Initially oriented images. The corresponding characteristic areas were marked in colors.

According to the previously mentioned methods for SAR image despeckling, the ASIFT algorithm was examined to verify its usability in the considered problem. The following scene presented in Figure 5 is considered.The SAR image being considered was gathered during one of the measurement campaigns in which the authors took part. During the raw data acquisition, the GPS data were stored together with the IQ signal samples. The optical image is the reference orthophotomap obtained during the geodetic measurements, and is defined by precise latitude and longitude information. As can be seen, a significant shift is visible in the SAR image, which has to be eliminated using the proposed approach. In theimage, corresponding regions were marked in colors, provingdirect georeference errors between the images. Next, the six described filters were employed to obtain speckle noise reduction, and the results were compared to the original SAR image.

**Figure 6:**
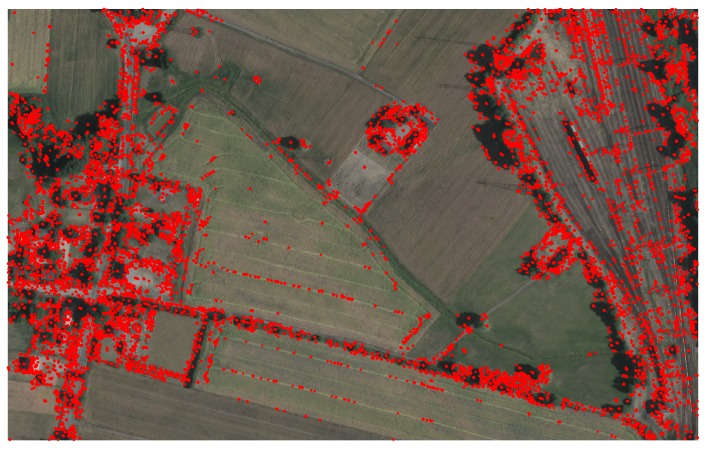
Orthophotomap with marked keypoints processed using the ASIFT algorithm.

In the process providing point cloud matching, two images are considered. The first is the orthophotomap, which is the same for all cases. The second image was changed to verify the proposed filters. An optical reference image is presented in Figure 6. The total number of keypoints found on the orthophotomap is 126,164.

### 5.1. Original SAR Image

**Figure 7:**
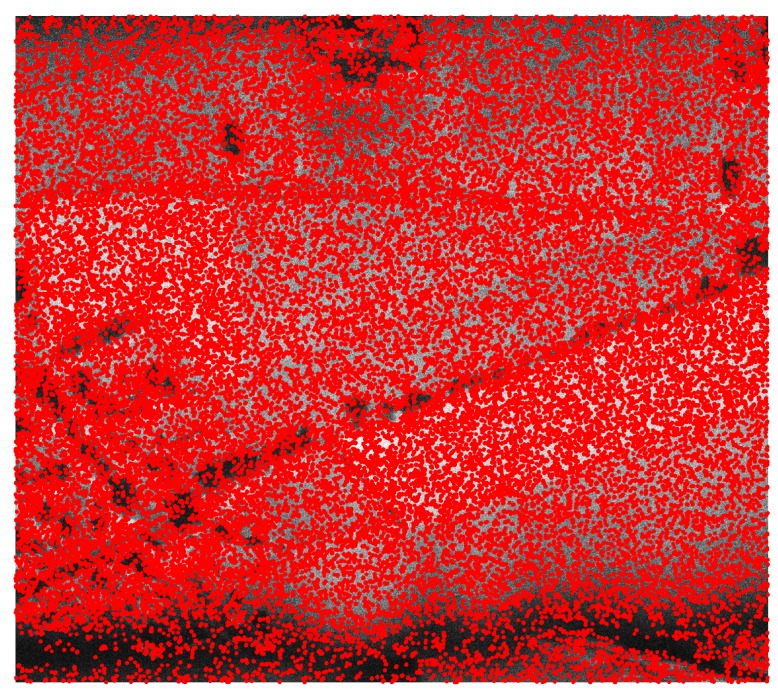
The results of the ASIFT algorithm processing for the initial SAR image.

In the first step, the original SAR image was considered. As mentioned, speckle noise may have significant influence when SAR data are processed in a typical way, ignoring this phenomenon. However, the problem under consideration requires additional processing, because such noise provides unwanted keypoints related to the nature of SAR image creation, not actual artifacts in the image. Figure 7 presents the results of the ASIFT algorithm processing for the initial SAR image.

**Figure 8:**
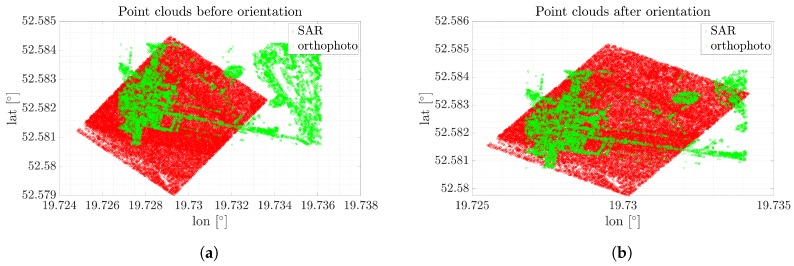
Point clouds before and after correction for the orthophotomap and original SAR image. (**a**) Point clouds of the SAR and optical images before orientatio, (**b**) Point clouds of the SAR and optical images after orientation.

As can be seen, speckle noise has a significant impact on the number of keypoints as well as their quality. The ASIFT algorithm found 367,584 keypoints, which is almost three times more in comparison to the optical image. Since speckle noise is present in all of the dataset, the distinguishing of the characteristic components or objects is impossible. However, the point clouds assigned to the images were processed, and the results before and after the operation are presented in Figure 8.

Because of the distorted data, it is impossible to find orientation correction. Because of the speckle noise, keypoints were found in the entire image, whereas the optical image is covered by keypoints only in the regions with the highest dynamic. As such, point clouds corresponding to keypoints in both images are correlated incorrectly.

**Figure 9:**
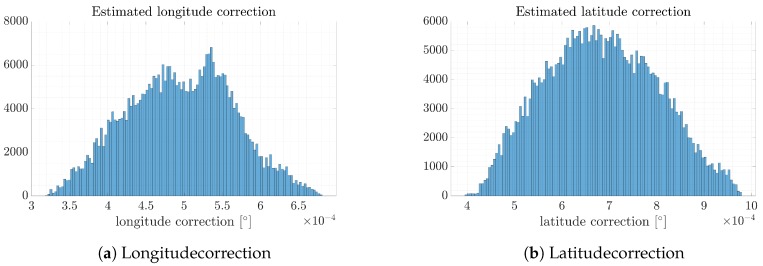
Longitude and latitude correction calculated based on the original SAR image.

For the analyzed point clouds, estimated latitude and longitude correction were calculated as a maximum value of histograms according to each coordinate. The histograms were performed by analyzing the shift of each point from the SAR image to match the optical reference orthophotomap. The results obtained are depicted in Figure 9. The estimated longitude correction is $\Delta_{\Leftrightarrow }=6.625\cdot 10^{-4}{[}^{\circ }]$ and the latitude correction is $\Delta_{⇕}=6.45\cdot 10^{-4}{[}^{\circ }]$. The results are compared with those obtained for despeckled images below.

### 5.2. SAR Image Filtered Using ML2D

**Figure 10:**
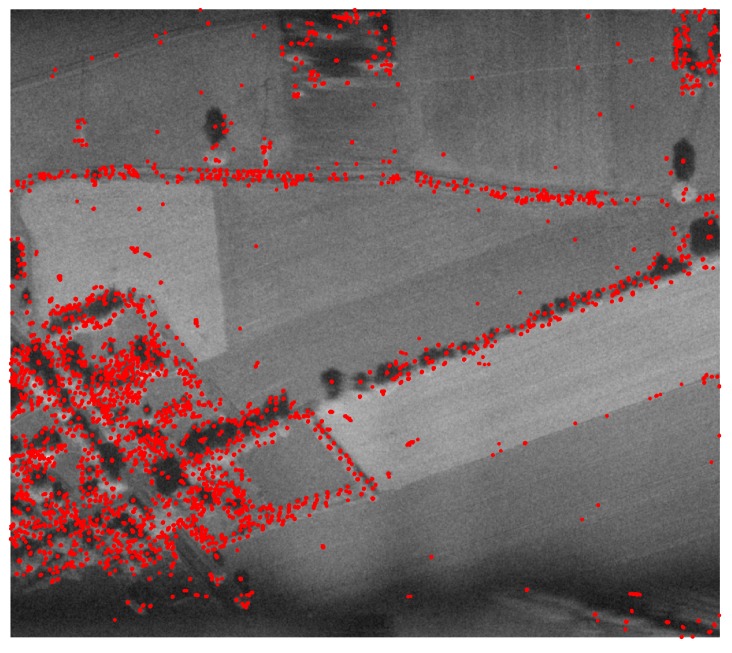
The results of the ASIFT algorithm processing for the SAR image filtered using ML2D method.

The ML2D method is presented in Section 4.1. Here, the results of the modified ASIFT algorithm are delivered. First, the keypoints were found in the filtered SAR image. The results obtained are depicted in Figure 10.

**Figure 11:**
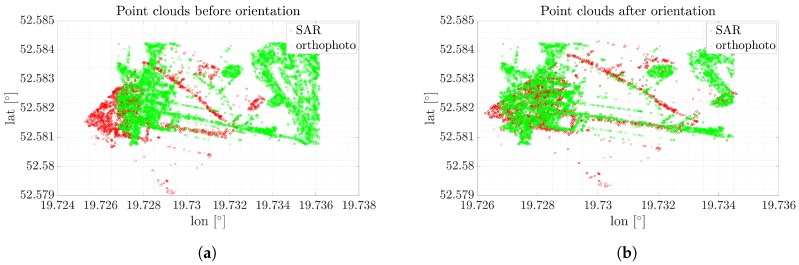
Point clouds before and after correction for the orthophotomap and SAR image filtered using ML2D method. (**a**) Point clouds of the SAR and optical images before orientation, (**b**) Point clouds of the SAR and optical images after orientation.

As can be noticed, the number of keypoints is significantly reduced by using the despeckling method. After this operation, keypoints were found in characteristic places such as trees, buildings, or roads. The ASIFT algorithm found 49,153 keypoints. The point clouds before and after orientation are presented in Figure 11.

**Figure 12:**
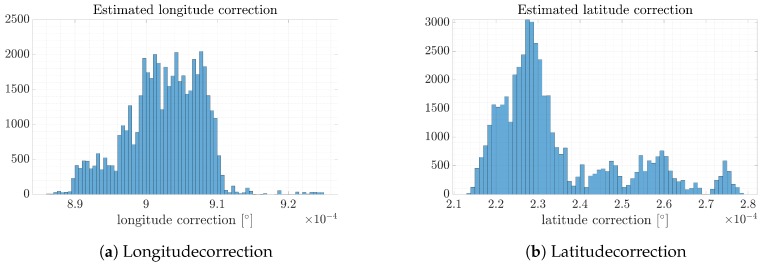
Longitude and latitude correction calculated based on the original SAR image filtered using ML2D method.

As expected, the filtered image allowed precise results to be obtained. Contours provided by the keypoints in the SAR scene were matched to the same scene, but illustrated using an optical sensor. Thanks to the ML2D method, the correction was estimated. Each coordinate is presented in Figure 12.

The estimated longitude and latitudecorrections are, respectively: $\Delta_{\Leftrightarrow }=9.775\cdot 10^{-4}{[}^{\circ }]$, $\Delta_{⇕}=2.69\cdot 10^{-4}{[}^{\circ }]$. It is worth noting that the character of histograms is different in comparison to the unfiltered data. In the initial case, the error had a Gaussian curve shape. After filtration, the latitude error can be clearly indicated. In the longitude case, the error is ambiguous. This is caused by the character of the SAR and optical image creation. Orthophotomaps are usually made perpendicular (NADIR) to the Earth, whereas SAR radars working in the StripMap mode illuminate scenes from a certain angle (off-NADIR). This dependency provides additional affine transformation, “stretching” an image in the direction of the antenna main lobe. However, by approximating the histogram using a Gaussian curve and interpolating data, the value corresponding to the correction can be estimated in a more precise way than in the original SAR image. Additionally, the correction sets are significantly narrower in comparison to the original data.

The results obtained for the other filters are similar and depicted in the same way. To make the text perspicuous, graphical representations such as keypoint clouds, histograms, and SAR images with marked points are attached in Appendix A.

### 5.3. SAR Image Filtered Using MEAN Filter

**Figure A1:**
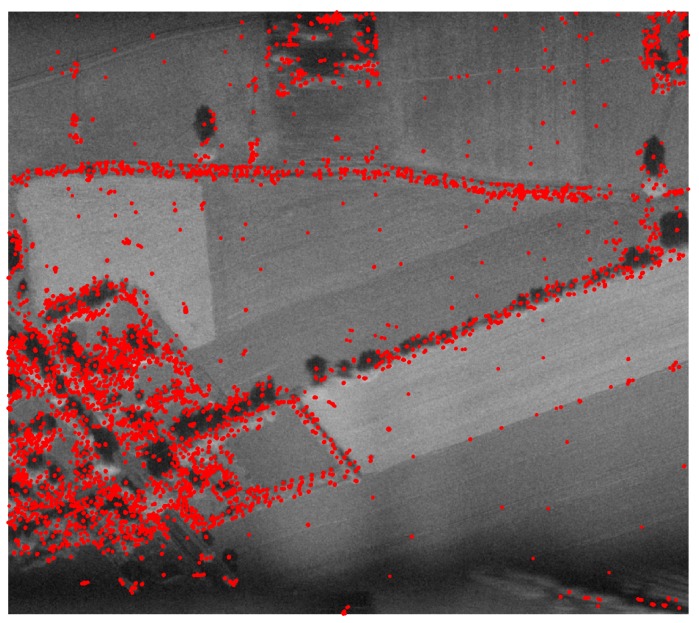
The results of the ASIFT algorithm processing for the SAR image filtered using MEAN method.

The next considered filter is presented in detail in Section 4.2. This approach is the simplest and probably the most intuitive, and can be quickly implemented to obtain a SAR image with reduced speckle noise. The filtered image with marked keypoints is depicted in Figure A1.

**Figure A2:**
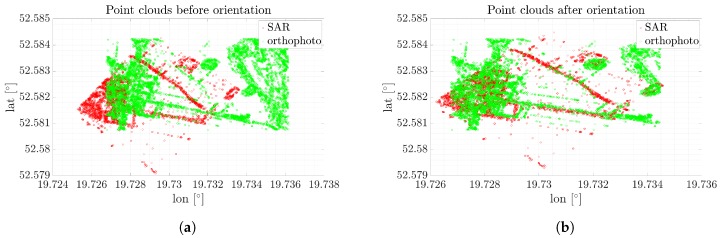
Point clouds before and after correction for the orthophotomap and SAR image filtered using MEAN filter (**a**) Point clouds of the SAR and optical images before orientation, (**b**) Point clouds of the SAR and optical images after orientation.

The number of detected keypoints significantly decreased. Despite the simple nature of the filter, the navigation error was estimated. The ASIFT algorithm detected 65,489 points whose distribution focused on characteristic places such as trees, buildings, etc. It enabled keypoints clouds obtained for both optical and SAR images to be comparable, as presented in Figure A2.

**Figure A3:**
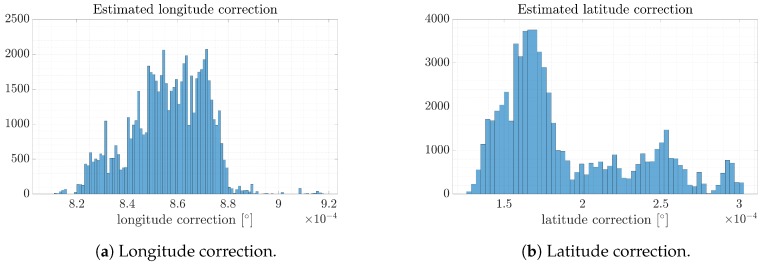
Longitude and latitude correction calculated based on the original SAR image filtered using ML2D method.

The MEAN filter allows improvement of the results to be obtained. According to the histograms presented in Figure A3, the corrected latitude and longitude are, respectively, $\Delta_{\Leftrightarrow }=9.465\cdot 10^{-4}{[}^{\circ }]$ and $\Delta_{⇕}=2.595\cdot 10^{-4}{[}^{\circ }]$. The presented histograms show the correction distribution, based on which the navigation drift is obtainable.

Figures provided for this subsection are attached in Appendix A.1.

### 5.4. SAR Image Filtered Using MMSE Filter

**Figure A4:**
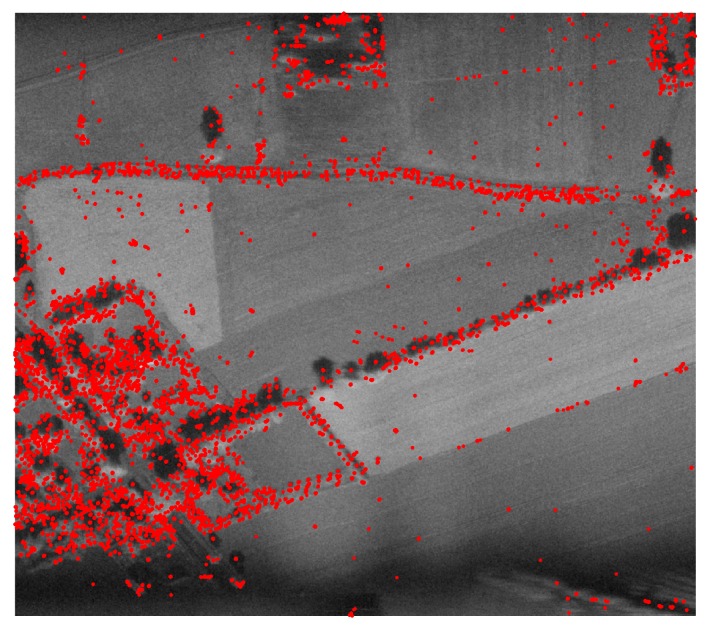
The results of the ASIFT algorithm processing for the SAR image filtered using MMSE filter.

The MMSE filter, described in Section 4.3, was examined as the next method for speckle noise reduction. As in the previous methods, in the first step, keypoints were detected using the ASIFT method, as presented in Figure A4.

**Figure A5:**
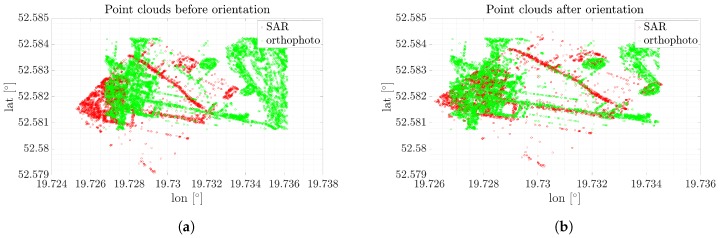
Point clouds before and after correction for the orthophotomap and SAR image filtered using MMSE filter. (**a**) Point clouds of the SAR and optical images before orientation, (**b**) Point clouds of the SAR and optical images after orientation.

As can be seen, a larger number of points was detected. The algorithm extracted 66,917 keypoints, which is clearly a higher value than in the previous results achieved for the algorithms of speckle noise reduction. By analyzing the result, it can be seen that points were found in places where they were not detected for other noise reduction methods. Keypoints are visible in fields and uniform areas, resulting in poorer point cloud quality. However, the overall point cloud structure has been preserved and correctly covered, as illustrated in Figure A5.

Although the quality of the keypoints is worse, because more points were detected, it was possible to correctly cover the characteristic areas distinguished from the images. A characteristic triangle formed by tree lines and buildings located in the left part of the picture were covered, determining the error being sought.

**Figure A6:**
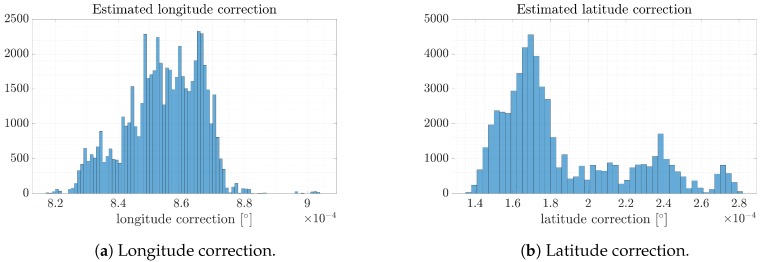
Longitude and latitude correction calculated based on the original SAR image filtered using MMSE filter.

The calculated longitude and latitude corrections presented in Figure A6 are as follows: $\Delta_{\Leftrightarrow }=9.355\cdot 10^{-4}{[}^{\circ }]$ and $\Delta_{⇕}=2.57\cdot 10^{-4}{[}^{\circ }]$. This is a similar result to the previously used methods. Accuracy is mainly limited by the histogram resolution, which can be improved by interpolation. Despite the detection of a larger number of points, which resulted from a smaller reduction of the speckle noise, it successfully allowed the detected keypoints clouds to be covered, and thus the navigation correction to be recalculated. The results are consistent with the methods presented above.

Figures provided for this subsection are attached in Appendix A.2.

### 5.5. SAR Image Filtered Using ELEE Filter

**Figure A7:**
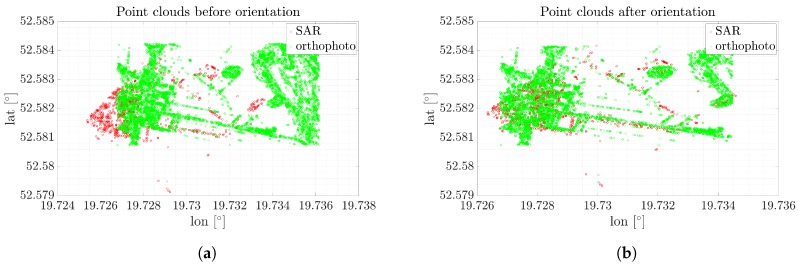
The results of the ASIFT algorithm processing for the SAR image filtered using ELEE filter.

The ELEE filter described in detail in Section 4.4 was employed as the next method for the improvement of point clouds covering. As in the case of the MMSE filter, more points were found both in places with increased dynamics and on uniform surfaces. This indicates less noise reduction; however, the overall character of the keypoint cloud was set as in the case of the MMSE filter. The results obtained are presented in Figure A7.

**Figure A8:**
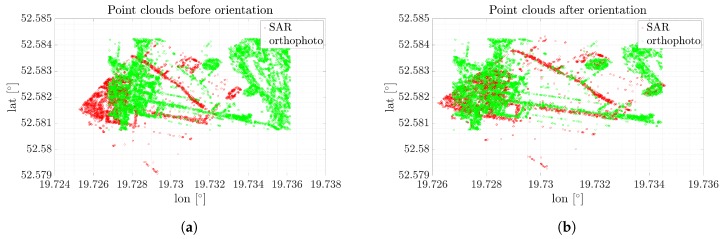
Point clouds before and after correction for the orthophotomap and SAR image filtered using ELEE filter. (**a**) Point clouds of the SAR and optical images before orientation, (**b**) Point clouds of the SAR and optical images after orientation.

For the discussed method, 58,165 characteristic points were detected, which is also a significant number compared to the analyzed methods. The keypoint clouds before and after the correction are shown in Figure A8.

**Figure A9:**
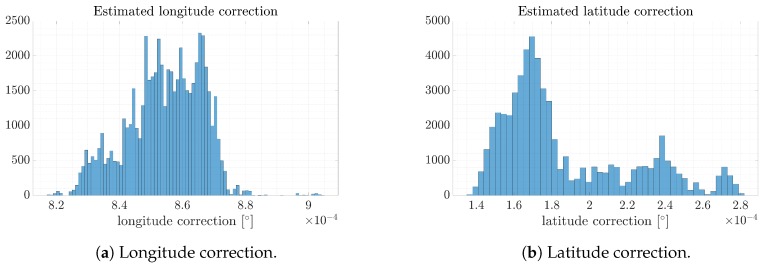
Longitude and latitude error calculated based on the original SAR image filtered using ELEE filter.

Again, the correction was calculated correctly, as evidenced by the coverage of characteristic areas in the images under investigation. It turns out that the method of point cloud shift correction is resistant to such small discrepancies and additional detection of characteristic points resulting from limited filtration of speckle noise. Histograms presenting navigation correction are presented in Figure A9. The estimated longitude and latitude corrections are, respectively: $\Delta_{\Leftrightarrow }=9.4175\cdot 10^{-4}{[}^{\circ }]$ and $\Delta_{⇕}=2.19\cdot 10^{-4}{[}^{\circ }]$.

Figures provided for this subsection are attached in Appendix A.3.

### 5.6. SAR Image Filtered Using GMAP Filter

**Figure A10:**
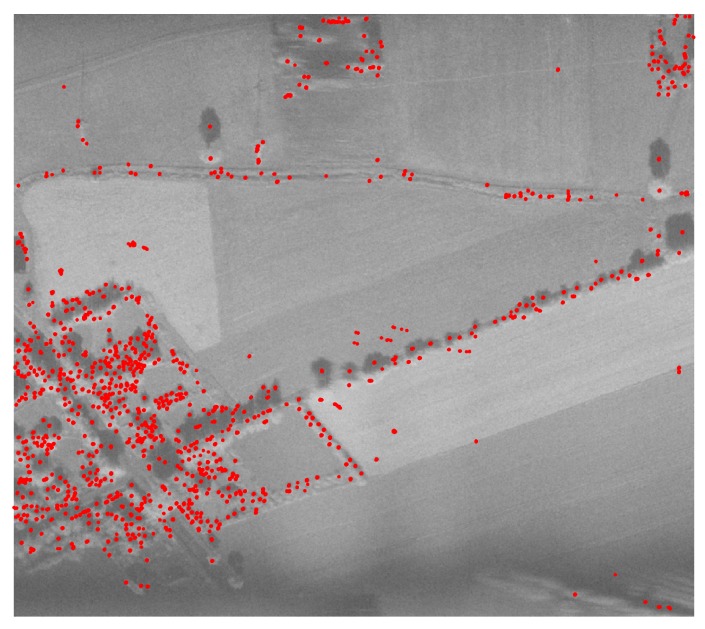
The results of the ASIFT algorithm processing for the SAR image filtered using GMAP filter.

The next investigated filter is GMAP, whose extended characteristic is presented in Section 4.5. The result showing the point cloud is illustrated in Figure A10.

The total number of points found in the image is 22,200, which is the smallest value for all of the considered cases. The algorithm detected characteristic points corresponding to different objects in the scene, which indicates a significant reduction in noise in the image.

**Figure A11:**
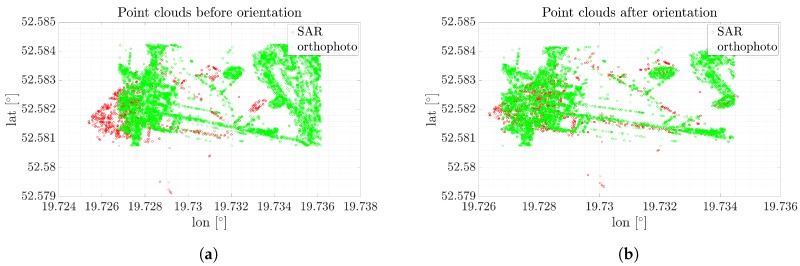
Point clouds before and after correction for the orthophotomap and SAR image filtered using GMAP filter. (**a**) Point clouds of the SAR and optical images before orientation, (**b**) Point clouds of the SAR and optical images after orientation.

**Figure A12:**
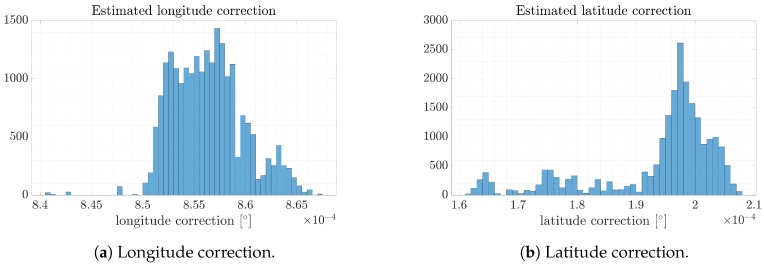
Longitude and latitude error calculated basing on the original SAR image filtered using GMAP filter.

**Figure A13:**
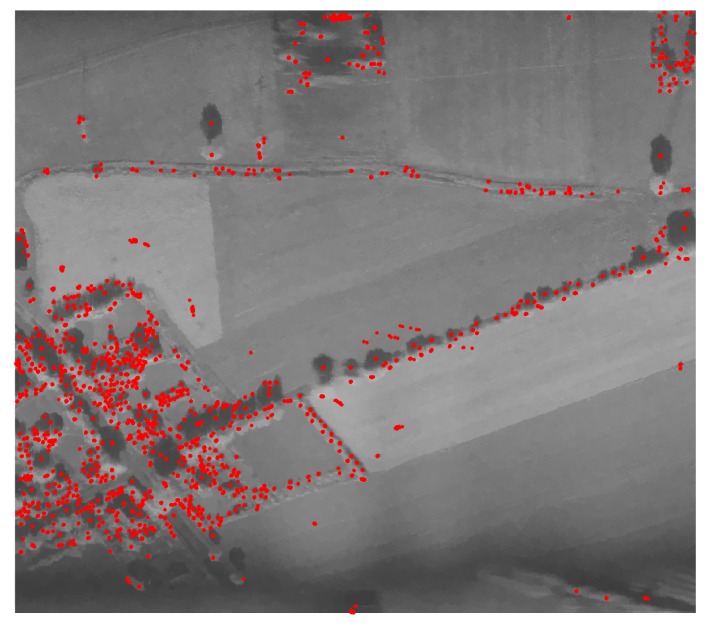
The results of the ASIFT algorithm processing for the SAR image filtered using SAR-BM3D filter.

As shown in Figure A11, the last filter also provides correct results. The point clouds are similar to those previously presented, making the GMAP filter an effective tool in the proposed method. It is worth noting that, for the considered image, there are a few regions where the resolution is low, such as in the lower part where trees are present, or in the upper part where buildings are depicted. Histograms presenting navigation correction are shown in Figure A12. Comparing the result in Figure A10 to the ones previously obtained, new details are available. In this case, the GMAP method shows a distinguishing object initially “hidden” in noise, which can be used in the point cloud analysis. The same effect is visible in Figure A13 for the SAR-BM3D filter. For the last considered case, the estimated longitude correction is $\Delta_{\Leftrightarrow }=9.635\cdot 10^{-4}{[}^{\circ }]$, whereas the estimated latitude correction is $\Delta_{⇕}=2.445\cdot 10^{-4}{[}^{\circ }]$, which correspond to the previously obtained values.

Figures provided for this subsection are attached in Appendix A.4.

### 5.7. SAR Image Filtered Using SAR-BM3D Filter

The last examined filter is BM3D, described in detail in Section 4.6. The same high resolution SAR image was processed to obtain a keypoint cloud, allowing navigation the correction to be estimated. The input image with marked keypoints is presented in Figure A13.

**Figure A14:**
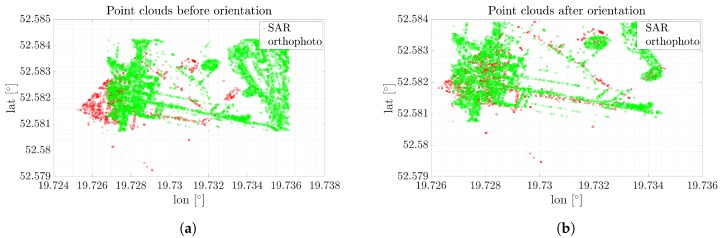
Point clouds before and after correction for the orthophotomap and SAR image filtered using SAR-BM3D filter. (**a**) Point clouds of the SAR and optical images before orientation, (**b**) Point clouds of the SAR and optical images after orientation.

The utilized despeckling method significantly improved the image quality (by reducing the noise level), which significantly affected the focusing of the detected keypoints in the most dynamic regions. The keypoints distribution corresponds to the results obtained for the different despeckling methods previously presented (including the number of detected keypoints). However, fewer keypoints were found in comparison to the previously presented outcomes (apart from the GMAP filter). The algorithm detected 24,954 points. In Figure A14, the keypoint clouds before and after orientation are presented.

**Figure A15:**
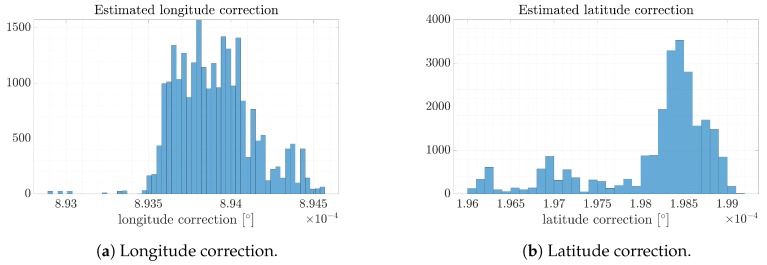
Longitude and latitude correction calculated based on the original SAR image filtered using SAR-BM3D filter.

Thanks to the reduction of speckle noise, negligible improvement was obtained, which affects the unequivocal shift estimation. The correction distribution, presented in Figure A15, is similar to the previous methods. In addition, in this case, the considered set is narrower than it was presented for the original SAR image without despeckling methods. The estimated longitude and latitudecorrections are, respectively: $\Delta_{\Leftrightarrow }=9.865\cdot 10^{-4}{[}^{\circ }]$$\Delta_{⇕}=2.475\cdot 10^{-4}{[}^{\circ }]$. This result proves the usability of such a filter for both, despeckling purposes as well as for the proposed modified ASIFT algorithm.

Figures provided for this subsection are attached in Appendix A.5.

### 5.8. Discussion

Each of the applied filters allowed estimationcorrections to be calculated in accordance with the assumptions. Due to the different nature of the creation of optical images and SAR, some imperfections are visible, but they are negligible in the analyzed problem. The filters processing performance is presented in Section 4.7. Table 2 shows the processing time using the ASIFT algorithm (does not consider filtering time) and the point cloud correlation. Additionally, the results of the correction estimation, as well as the number of points found in each of the images, are summarized.

As can be seen, similar results were obtained for all filters tested. The outcomes of the rotation estimation are characterized by the following relationship: the greater is the number of points found, the greater is the rotation correction determined. Interestingly, the calculation time is not strongly dependent on the number of keypoints detected. However, storing points requires more memory, and, even if the calculation time is similar, the memory required is larger for the method detecting a greater number of points. It should be noted that all computation was performed on the CPU without any parallelization. To decrease the computation time and boost the computation speed, a GPU should be used.

The experiments proved that the proposed method for the geometrical matching of SAR and optical images utilizing ASIFT features for SAR-based Navigation Aided Systems is suitable for compute the corrections to the SAR images’ direct georeferencing. In the literature, there are many methods and algorithms for this type of data co-registration [9,11,12,13,15,16,18,19]. All of these algorithms were tested on SAR images and optical images acquired from space. The proposed methodology of data integration is based on high resolution (in full resolution) SAR images and orthophotomaps obtained from altitude. Thus, it is hard to compare the presented method with the method described in [9,11,12,13,15,16,18,19], because of the spatial resolution of satellite optical images and the size of overlapping areas. For this reason, it required guaranteeing well-distributed (in the whole investigation area), robust corresponding points. To achieve this, a modification of the SfM approach based on the ASIFT detector and he elimination of the keypoints description, as well as description matching step and using the two-step ICP method, was performed. To compute the transformation parameters, methods based on finding the pairs of points are used. This way of determining corresponding points is useful when the points are well distributed throughout the overlapping areas. Unfortunately, when data from attitude are processed, this relationship may not be met and the matching pairs of points will not be evenly distributed throughout the study area. This might cause wrong or inaccurate determination of correction parameters. Therefore, the use of the ASIFT algorithm allows the detection of more points evenly distributed throughout the entire area of work. In connection with the ICP method, the keypoints will not be aligned in pairs, but will form a rigid body that reduces the influence of the outliers on the final determining transformation elements.

**Figure 13:**
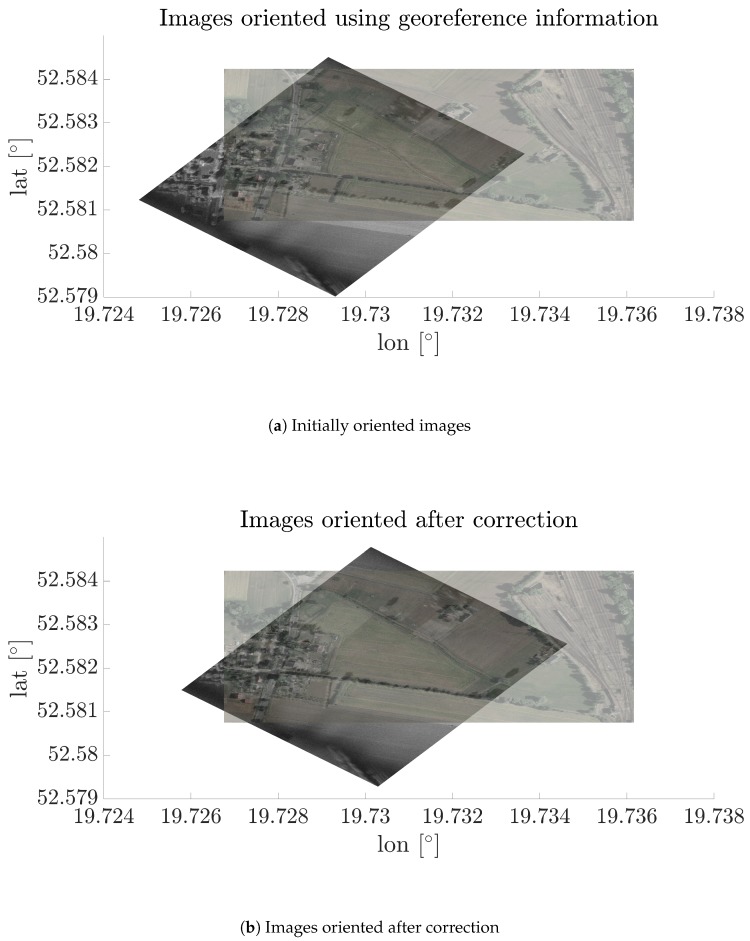
Oriented images before and after correction.

In Figure 13, the oriented SAR and optical images before and after correction are presented. In this case, correction calculated by the ELEE method was utilized, however the results are comparable for all cases when despeckling filters are used. As can be seen, characteristic objects such as trees, roads, and buildings are covered.

On the basis of the results obtained, the calculated correction value per kilometer can also be estimated. Assuming that $1^{\circ }\approx 111.1$ km, the longitude correction is about 105 m, while for latitude the correction was about 25 [m]. This is a significant value, especially in the case of military systems for which the required precision should be as high as possible. For the case where the despeckling filter was not applied, the navigation correction is inappropriate and amounts to 73.6 m for longitude and 71.6 m for latitude, which are the mean values of the differences between the original coordinates of the SAR and optical images taken into consideration. This shows how important it is to combine the presented methods and implement the processing pipeline. The juxtaposition also shows that, from the point of view of the implementation of navigation correction on a flying platform, it is sufficient to use simple despeckling filters, which can significantly reduce computational effort and thus accelerate processing, which is particularly important for fast flying objects. It should be noted that the final resolution of the correction is limited by the radar bandwidth, for which the range resolution is expressed by the relationship $\delta R=\frac{c}{2B}$, where *c* is the speed of light and *B* is radar signal bandwidth. For the proposed method to make sense, high resolution radars should be used, which, unfortunately, are associated with a greater financial outlay and a requirement for high processing power of the computing unit. In addition, it should be taken into account that the obtained high resolution radar images must be filtered to reduce speckle noise, which limits the resolution of the entire imaging. Moreover, phenomena such as heterogeneous movement of the radar carrier platform or changing its speed during the flight in an unpredictable way also degrade the quality of the image. Nevertheless, taking into account and minimizing such phenomena, it is possible to use the method proposed by the authors, which was proved experimentally.

## 6. Conclusions

This paper presents a novel algorithm for navigation correction estimation dedicated to flying platforms (e.g., drones, airplanes, cruise missiles, etc.) in cases when satellite navigation systems are unavailable. This method utilizes various techniques to:

- filter SAR images;
- find keypoints on SAR and optical images;
- find shift and rotation correction between images; and
- calculate navigation correction between an acquired SAR image and a reference optical image.

Combining such methods in a single solution is a novel approach proposed by the authors. Taking advantage of several techniques, an innovative algorithm was proposed, tested and validated to confirm its usability for SAR images obtained during a measurement campaign. This solution may be useful in military and civilian applications, when the lack of a GNSS signal is a critical problem which makes flying missions impossible. Merged techniques such as ASIFT-based keypoint extraction and SfM-based keypoints matching make this method robust and resistant to noise and interference. Thus, the presented methodology can be successfully integrated with existing systems to enhance their precision and dependability. Additionally, the authors provide a comparison of several filters, including their computational complexity and performances. This presents a wide variety of uses for this technique depending on the solution. In the future, the authors intend to implement the method on a real-time platform and test and verify the proposed methodology in real conditions, which should confirm its usability. The target platform is the GPU (Graphics Processing Unit), allowing fast computing to be obtained (both SAR processing and correction estimation). Another crucial issue is the implementation of the functionality that would be able to cope with the situation where the imaged scene has been significantly changed compared to the one that was saved in the database (as an optical image). Such a situation may arise when, for example, the imaged area has been damaged as a result of a disaster or war, and the database on board the flying object does not have current pictures. The estimation may then be ineffective, which is undesirable. This is currently the main problem the authors are working on.

## Figures and Tables

**Table 1:** SAR^†‡^ images filtration performance comparison.

Filter Type	ENL\ENIL	Processing Time
Ml2d	23.9	12 s
Mean	21.7	23 s
Mmse	17.9	43 s
Elee	19.3	03 m:25 s
Gmap	24.1	03 m:28 s
Sar-Bm3d	199.2	04 h:05 m:17 s

† *Original SAR image size 2540×2250[px];* ‡ *Original SAR image ENL=1.*

**Table 2:** Estimated coordinates correction for different filters.

Filter Type	Longitude Correction ${[}^{\circ }]$	Latitude Correction ${[}^{\circ }]$	Rotation ${[}^{\circ }]$	Processing Time [*s*]	Amount of Keypoints
No filter	$6.6250\times 10^{-4}$	$6.4500\times 10^{-4}$	4.0919	111.7241	367,584
ML2D	$9.7750\times 10^{-4}$	$2.6900\times 10^{-4}$	0.9416	79.9240	49,153
MEAN	$9.4650\times 10^{-4}$	$2.5950\times 10^{-4}$	0.5612	71.8246	65,489
MMSE	$9.3550\times 10^{-4}$	$2.5700\times 10^{-4}$	0.6804	77.3086	66,917
ELEE	$9.4175\times 10^{-4}$	$2.1900\times 10^{-4}$	1.2547	71.1760	58,165
GMAP	$9.6350\times 10^{-4}$	$2.4450\times 10^{-4}$	0.0051	66.2229	22,200
BM3D	$9.8650\times 10^{-4}$	$2.4750\times 10^{-4}$	0.3204	66.9932	24,954

## Appendix A

In this appendix, the results obtained in Section 5 are presented.
